# Optimal pace timing for left bundle branch area pacing with or without an additional LV lead: results from the CSPOT study

**DOI:** 10.1093/europace/euag103

**Published:** 2026-05-05

**Authors:** Robert D Schaller, Paul Foley, Badrinathan Chandrasekaran, Jonathan Lyne, Marek Jastrzębski, Pugazhendhi Vijayaraman, Gaurav A Upadhyay, Rafał Gardas, Travis Richardson, D’Anne Kudlik, Robert W Stadler, Patrick Zimmerman, James Burrell, Robert Waxman, Richard N Cornelussen, Bengt Herweg, Zachary Whinnett

**Affiliations:** Section of Cardiac Electrophysiology, Cardiovascular Division, Department of Medicine, Hospital of the University of Pennsylvania, 3400 Civic Center Boulevard, Philadelphia, PA 19104, USA; Wiltshire Cardiac Center, Great Western Hospital, Swindon, UK; Wiltshire Cardiac Center, Great Western Hospital, Swindon, UK; Division of Cardiac Electrophysiology, Beacon Hospital, Dublin, Ireland; First Department of Cardiology, Interventional Electrocardiology and Hypertension, Jagiellonian University, Medical College, Krakow, Poland; Geisinger Heart Institute, Geisinger Commonwealth School of Medicine, Wilkes-Barre, PA, USA; Center for Arrhythmia Care, Section of Cardiology, University of Chicago, Pritzker School of Medicine, Chicago, IL, USA; Department of Electrocardiology and Heart Failure, Medical University of Silesia, Katowice, Poland; Division of Cardiovascular Medicine, Vanderbilt University Medical Center, Nashville, TN, USA; Medtronic, Mounds View, MN, USA; Medtronic, Mounds View, MN, USA; Medtronic, Mounds View, MN, USA; Medtronic, Mounds View, MN, USA; Medtronic, Mounds View, MN, USA; Medtronic, Bakken Research Center, Maastricht, Netherlands; Division of Cardiology, University of South Florida Morsani College of Medicine and Tampa General Hospital, Tampa, FL, USA; National Heart and Lung Institute, Imperial College, London, UK

**Keywords:** cardiac resynchronization therapy, Left bundle branch area pacing, Left bundle branch area pacing-optimized cardiac resynchronization therapy, AV delay, VV delay

## Abstract

**Aims:**

This study aimed to determine optimal atrioventricular (AV) timing for left bundle branch area pacing (LBBAP) and optimal AV and interventricular (VV) timing for LBBAP optimized cardiac resynchronization therapy (LOT-CRT) to enhance the benefit of conduction system pacing.

**Methods and results:**

Acute measurements of LV dP/dt_max_ and QRS duration were collected during sweeps of AV and VV delays during paired comparison of LBBAP, LOT-CRT, and biventricular pacing (BVP) in 48 CRT-indicated patients. Optimal AV delays were selected as the vertex of a parabolic model fit to the AV sweep results, obtained independently for LV dP/dt_max_ and QRS duration. LBBAP, LOT-CRT, and BVP were similarly sensitive to the choice of AV delay, and 60 ms deviations from optimal AV delay resulted in an approximately 30% reduction in the haemodynamic benefits of resynchronization. Based upon LV dP/dt_max_ measurements, the average optimal paced AV (PAV) for LBBAP (152 ms) was significantly shorter than that for LOT-CRT (171 ms, *P* < 0.01) or BVP (167 ms, *P* = 0.01). Based upon QRS duration, the average optimal PAV for LBBAP (164 ms) was similar to that for LOT-CRT (160 ms, *P* = 0.52) or BVP (165 ms, *P* = 0.95). For each resynchronization modality, a linear regression between the patient's measured intrinsic AV conduction and their optimal AV delay provided a patient-specific AV delay, resulting in improved AV selection compared to applying a fixed AV delay to all patients. Finally, simultaneous activation (VV = 0) was best for both LOT-CRT and BVP.

**Conclusion:**

LBBAP, LOT-CRT, and BVP are similarly sensitive to AV-timing. Optimal AV delay is linearly related to intrinsic AV for each resynchronization modality. For LOT-CRT and BVP, simultaneous biventricular activation is best on average.

**Clinical Trial Registration information:**

https://clinicaltrials.gov/study/NCT04905290

## Graphical Abstract

**Graphical Abstract:**
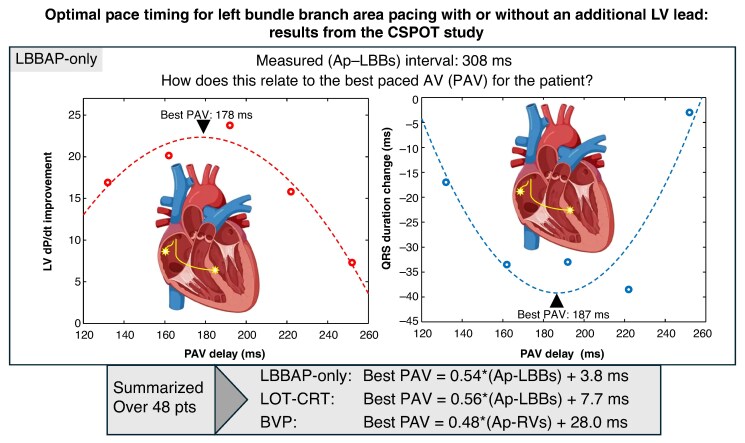

What's new

- We present the first direct, within-patient comparison of AV and VV delay sensitivity and optimization among LBBAP, LOT-CRT, and BVP, utilizing both acute haemodynamic (LV dP/dt_max_) and electrical (QRS duration) endpoints.
- By applying a parabolic model to AV delay sweeps, we identified optimal AV delays for each pacing modality, demonstrating that a 60 ms deviation from optimal AV timing leads to a ∼30% reduction in haemodynamic benefit, underscoring the clinical importance of precise timing programming.
- We further show that optimal AV delay is linearly related to intrinsic AV conduction, allowing individualized delay selection through regression modelling. This approach outperforms fixed AV delays and could be readily implemented in clinical practice.
- Our data reveal that simultaneous biventricular activation (VV = 0 ms) is optimal for both LOT-CRT and BVP, providing practical guidance for device programming.

## Introduction

Cardiac resynchronization therapy (CRT) using biventricular pacing (BVP) is well established, with numerous reports addressing the importance of optimizing atrioventricular (AV) and interventricular (VV) timing.^1–3^ One mechanism by which CRT improves cardiac output is by improving left ventricular filling by optimizing the timing of atrial and ventricular activation.^4^ Appropriate AV timing during BVP is essential to reduce dyssynchrony between the atria and ventricles, and appropriate VV timing is important to reduce dyssynchrony between the left and right ventricles. Left bundle branch area pacing (LBBAP)^5–9^ and the combination of LBBAP with traditional CRT via coronary venous pacing (LOT-CRT)^10,11^ have recently emerged as alternative approaches to BVP. However, optimization of AV timing in LBBAP and both AV and VV timing in LOT-CRT remain largely unexplored.

We previously evaluated patients with non-specific interventricular conduction delay (IVCD) and left bundle branch block (LBBB) to compare acute changes during three approaches to resynchronization: BVP, LBBAP, and LOT-CRT.^12^ During implantation, LV dP/dt_max_ and QRS duration were measured in paired comparisons of the three approaches to resynchronization, including sweeps of AV and VV delays. The aim of this study was to evaluate the sensitivity of each approach to AV delay, explore the relationship between intrinsic AV interval and optimal AV delay, and identify the optimal VV delay for LOT-CRT.

## Methods

### Study design

The Conduction System Pacing Optimized Therapy (CSPOT) study (ClinicalTrials.gov NCT04905290) was a prospective, multicentre, global investigation of resynchronization pacing in CRT-indicated patients. The study design and population have been described previously.^12^ Briefly, patients with an indication for CRT were included, while those with complete heart block or atrial fibrillation (AF) were excluded. All participants underwent standard CRT implantation with the addition of an LBBAP lead.

All leads were acutely connected to a recording system (EP Tracer, Schwarzer-Cardiotek GMBH, Germany) for continuous electrogram (EGM) recording. LV pressure was recorded using a Millar catheter (Millar Inc., Texas, USA) along with a continuous 12-lead ECG. Atrial pacing was delivered at a constant overdrive rate to evaluate the acute haemodynamic response to three resynchronization strategies: LBBAP (unipolar to avoid anodal stimulation), LOT-CRT, and BVP. For each approach, five AV delays were tested: 70% of the interval between atrial pacing and the earliest QRS activation on 12-lead ECG (AV70%), AV70% +/−30 ms, and AV70% +/− 60 ms. For LOT-CRT, four VV delays were evaluated, all using AV70%: VV = 0, LBBAP first by 30 ms, LV first by 30 ms, and LV first by 60 ms. For BVP, three VV delays were tested, all using AV70%: VV = 0, LV first by 30 ms, and LV first by 60 ms.

The study adhered to the Declaration of Helsinki and received ethics committee approval at each site. All participants provided written informed consent.

### Extracted measurements

QRS duration was measured on the standard 12-lead ECG from the initial deflection from the isoelectric line to the end of the QRS complex. Measurements did not always begin at the pacing stimulus, as longer AV delays during testing occasionally allowed intrinsic ventricular activation to precede pacing. For each pacing configuration, the electrocardiographic response was defined as the absolute reduction in paced QRS duration compared to intrinsic activation.

For each tested AV and VV delay, the percentage change of LV dP/dt_max_, relative to that of intervening segments of AAI-only pacing, was measured during each of eight transitions (four segments of resynchronization pacing vs. five segments of AAI pacing), as described previously.^12^ Because all LV dP/dt_max_ changes were measured relative to neighbouring segments of AAI pacing, drift over the procedure duration should be minimized.

All datasets with completed AV sweeps for LBBAP, LOT-CRT, or BVP were included to assess the impact of AV delay selection. For each approach, we averaged the LV dP/dt_max_ and QRS duration across the AV sweep and calculated the range (maximum–minimum values) of LV dP/dt_max_ and QRS duration observed during the AV sweep.

**Figure 1:**
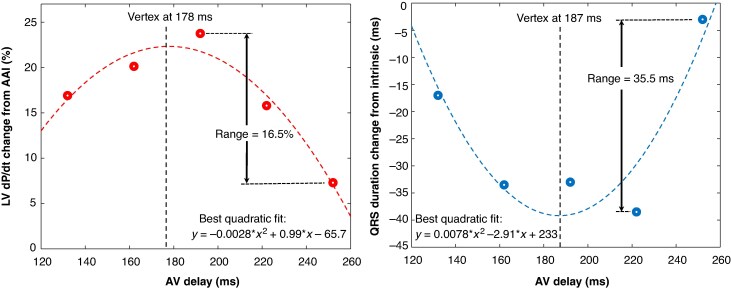
The results from a sweep of 5 AV delays during LBBAP in an example patient. Left: LV dP/dt spanned a range of 16.5% improvement over AAI pacing, and a parabolic fit suggested an optimal AV of 178 ms. Right: QRS duration reduction from intrinsic spanned 35.5 ms, and a parabolic fit suggested an optimal AV delay of 187 ms.

For each patient and resynchronization method, parabolic models were independently fit to LV dP/dt_max_ and QRS duration results across the five tested AV delays (*Figure 1***)**. The vertex of each parabola was used to identify the optimal AV delay for both LV dP/dt_max_ and QRS duration. This analysis included those patients whose AV sweep results were well modelled by a parabola. Specifically, the model fit had to exceed R^2^ = 0.5, the vertex of the parabola had to occur no further than 30 ms beyond the tested AV sweep, the parabolic fit had to produce a positive quadratic coefficient for QRS duration reduction and negative quadratic coefficient for LV dP/dt_max_ improvement, and the response to the AV sweep could not be ‘flat’ (i.e. the QRS duration measurements from the five AV delays had to span a range of at least 5 ms, and the LV dP/dt_max_ measurements from the five AV delays had to span a range of at least 3% change relative to that of AAI pacing). Patients whose data did not meet these criteria were excluded from this portion of the analysis.

Finally, we evaluated the correlation between each patient's optimal AV delay for resynchronization (separately for LBBAP, LOT-CRT, and BVP) and their intrinsic AV delay during atrial pacing (the study excluded patients with complete heart block or AF). Intrinsic AV conduction was estimated during atrial pacing by applying a simulated ventricular sense amplifier to the RV tip-ring and LBB tip-ring EGMs (MATLAB version 2024a, MathWorks, Inc.). The interval from an atrial pace to ventricular-sensed event (i.e. either Ap to RVs or Ap to LBBs) was measured for each patient.

### Statistical analysis

Baseline data were summarized using means and standard deviations for continuous data and counts with percentages for categorical data. Mixed models for repeated measures were used to analyse each combination of outcome (optimal AV delay and observed range) and acute measurement (LV dP/dtmax and QRS duration) with a patient-level intercept. Pacing modality comparisons were performed using Wald tests with Satterthwaite degrees of freedom. Multiple comparisons were adjusted using the Holm-Bonferroni method separately for optimal AV delay and observed range (6 comparisons each). Analysis was performed in R (R Core Team 2023 and ImerTest, Vienna, Austria).

## Results

### Patient characteristics

The 48 subjects that completed the study protocol have been described previously^12^ and are summarized in *Table 1*. The cohort was predominantly male (67%), with nearly one-third having ischaemic cardiomyopathy, and the mean LV ejection fraction was 30.6%. Centralized ECG adjudication classified 40% of patients with LBBB and 60% with IVCD, and classified successful LBBAP in 56% of patients and DSP capture in the remaining 44% of the patients.

**Table 1:** Patient demographics at implant

Characteristic	Total (*n* = 48)
Age (years)	65.3 ± 10.3
Male	32 (67%)
ICM	14 (29%)
LBBB	19 (40%)
LVESV (mL)	144.4 ± 61.8
LVEF (%)	30.6 ± 11
Intrinsic QRS duration (ms)	171.2 ± 20.8
Intrinsic PR interval (ms)	200.1 ± 53
LBBAP Quality	
Successful LBBAP	27 (56%)
DSP	21 (44%)

ICM, Ischemic cardiomyopathy; LBBB, left bundle branch block; LVESV, left ventricular end systolic volume; LVEF, left ventricular ejection fraction; ms, millisecond. Continuous data are represented as mean ± standard deviation, and binary data are represented as count (percentage).

### Sensitivity to AV delay

*Figure 1* illustrates the improvement in LV dP/dt_max_ and QRS duration during LBBAP for a representative patient. A 120 ms sweep of five AV delays was tested, centred around an AV70% of 192 ms (i.e. 70% of the patient's interval between atrial pacing and the earliest QRS activation on 12-lead ECG was 192 ms). The sweep yielded a 16.5% range in dP/dt_max_, with a quadratic fit (R^2^ = 0.94) indicating an optimal AV delay (i.e. the vertex of the parabola) of 178 ms. For QRS duration, the range was 35.5 ms, and the corresponding quadratic model (R^2^ = 0.85) identified an optimal AV delay of 187 ms.

**Figure 2:**
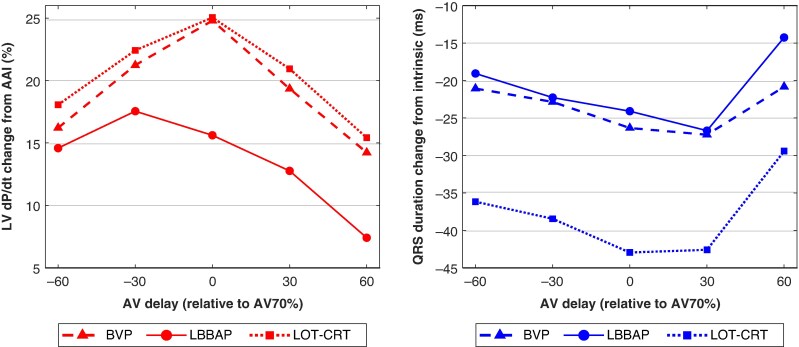
Left: the relationship between LV dP/dt and AV delay, averaged over all patients. Right: The relationship between QRS duration and AV delay, averaged over all patients.

*Figure 2* summarizes the average relationship for all patients between AV delay and both LV dP/dt_max_ and QRS duration across the three resynchronization strategies. The AV70% for each patient (174 ± 43 ms, range 122–372 ms) was normalized to zero in this Figure. As previously shown in this cohort, unipolar LBBAP produced a smaller improvement in LV dP/dt_max_ compared to BVP and LOT-CRT, while LOT-CRT achieved greater QRS narrowing than both LBBAP and BVP.^12^ However, the overall shape of the LV dP/dt_max_ and QRS-duration curves as functions of AV delay suggests a similar importance of AV optimization for the three resynchronization methods. Notably, an AV delay of 60 ms away from the optimal value reduced the LV dP/dt_max_ benefit by approximately 30%.

**Figure 3:**
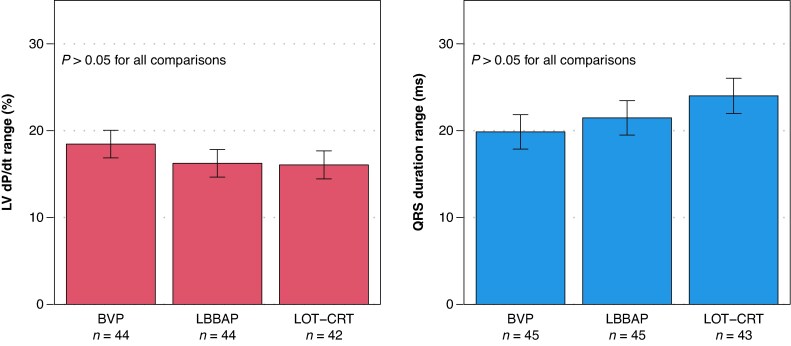
The three resynchronization methods (BVP, LBBAP, and LOT-CRT) produced similar ranges of LV dP/dt improvement (% change from AAI pacing) and QRS duration (ms reduction from intrinsic) during 120 ms sweeps in AV delay, suggesting similar sensitivity to selection of AV delay. Error bars represent the model-based estimate of the standard error of the mean.

Sensitivity to AV delay was further assessed by measuring the range (max–min) of LV dP/dt and QRS duration across the AV sweep from AV70% – 60 ms to AV70% + 60 ms. When averaged over all patients (*Figure 3*), the range of LV dP/dt did not differ significantly among BVP, LBBAP, and LOT-CRT (*P* = 0.22) nor did the range of QRS duration (*P* = 0.29). These findings further support that AV optimization is equally important across all three resynchronization strategies.

### Optimal AV delay

**Figure 4:**
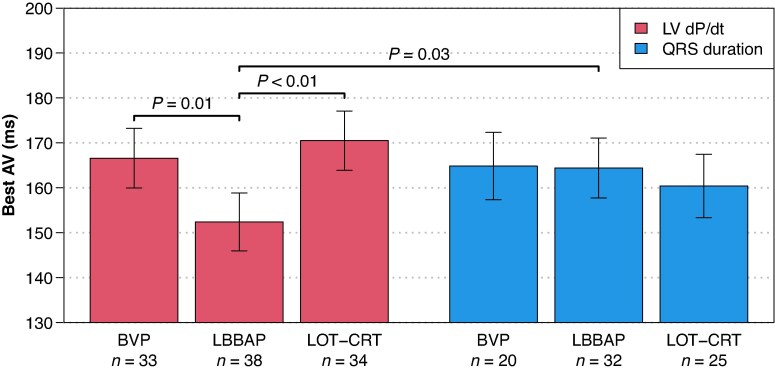
The best AV delay during atrial pacing, averaged over patients. For each patient, the best AV was obtained as the vertex of the best quadratic fit to the sequence of 5 tested AV delays. Error bars represent the standard error of the mean.

For each patient with an acceptable parabolic fit, the optimal paced AV delay (PAV) was determined separately based on LV dP/dt_max_ improvement and QRS duration reduction (*Figure 1*), with average values shown in *Figure 4*. A significant interaction was observed, indicating that the optimal PAV depended on both the pacing method and metric used (*P* = 0.02). When based on LV dP/dt_max_, the average optimal PAV for LBBAP (152 ms) was significantly shorter than for BVP (167 ms, *P* = 0.01) or LOT-CRT (171 ms, *P* < 0.01). When based on QRS duration, the optimal PAV for LBBAP (164 ms) was similar to that for BVP (165 ms, *P* = 0.95) or LOT-CRT (160 ms, *P* = 0.52). Notably, for LBBAP, the optimal PAV obtained by LV dP/dt_max_ (152 ms) was shorter than that determined by QRS (164 ms, *P* = 0.03), whereas no significant differences between metrics were observed for LOT-CRT (*P* = 0.09) or BVP (*P* = 0.79).

### Patient sub-populations

Multivariate analysis of optimal AV delay, incorporating covariates for conduction type (LBBB vs. IVCD), LBBAP capture type (LBBAP vs. deep septal pacing), and cardiomyopathy aetiology (ischaemic vs. non-ischaemic), revealed no statistically significant effects from these factors. The interaction between resynchronization method and resynchrony metric remained significant when these covariates were included (*P* = 0.02).

### Relationship between intrinsic AV and optimal paced AV

We next assessed whether the optimal AV delay for resynchronization pacing (BVP, LBBAP, or LOT-CRT) was correlated with the patient's intrinsic AV interval. Intrinsic AV conduction was measured from atrial paced event to ventricular sensed event on the RV EGM (for BVP) or LBBAP EGM (for LBBAP or LOT-CRT). In this cohort, the average Ap-RVs interval (279.0 ± 49.2 ms) was nearly identical to the Ap-LBBs interval (278.4 ± 48.1 ms, *P* = 0.85).

**Figure 5:**
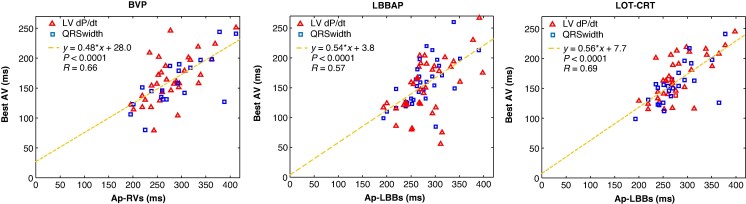
The relationship between each patient's Ap-RVs or Ap-LBBs interval and their best PAV for BVP (left), LBBAP (middle), and LOT-CRT (right) resynchronization strategies. The best PAV was determined independently for the maximum LV dP/dt and the minimum QRS duration, according to the vertex of a quadratic model applied to measurements during AV sweeps.

For patients with an acceptable parabolic fit, *Figure 5* compares the optimal PAV, determined independently by LV dP/dt and QRS duration, to the intrinsic AV interval measured as Ap-RVs (for BVP) or Ap-LBBs (for LBBAP and LOT-CRT). In the left panel, during BVP with zero VV delay, linear regression showed that optimal PAV for BVP could be estimated as 0.48*(Ap-RVs) + 28.0 ms (*P* < 0.0001). In the middle panel, during LBBAP, the best PAV was approximated by 0.54*(Ap-LBBs) + 3.8 ms (*P* < 0.0001). In the right panel, during LOT-CRT pacing with zero VV delay, the optimal PAV was approximated by 0.56*(Ap-LBBs) + 7.7 ms (*P* < 0.0001).

### Value of fixed AV delays

For each resynchronization method, the optimal fixed AV delay (i.e. a horizontal line in *Figure 5*) was defined as the mean of the best AV delays for the population. *Table 2* compares the performance of applying the best fixed PAV to all patients, applying the nominal device PAV (130 ms), and applying the patient-specific PAV based on the linear-regression equations from *Figure 5*.

**Table 2:** Comparison of approaches to select AV delay and the resulting mean error relative to the best AV for each patient

	Best fixed PAV	Mean error of fixed PAV compared to best PAV	Nominal PAV	Mean error of nominal PAV compared to best PAV	Patient-specific PAV (Linear regression)	Mean error of patient-specific PAV compared to best PAV
**BVP**	163 ms	33.0 ms	130 ms	40.7 ms	0.48*(Ap-RVs) + 28.0 ms	22.2 ms
**LBBAP**	155 ms	34.6 ms	130 ms	38.7 ms	0.54*(Ap-LBBs) + 3.8 ms	26.6 ms
**LOT-CRT**	163 ms	29.2 ms	130 ms	37.8 ms	0.56*(Ap-LBBs) + 7.7 ms	19.3 ms

The patient-specific PAV approach (i.e. linear regression) is from ***Figure 5***.

For LBBAP-only, the nominal PAV (130 ms) was more than 30 ms away from the optimal PAV for 55.3% (21/38) of patients according to LV dP/dt_max_, and more than 30 ms away from the optimal PAV for 46.9% (15/32) of patients according to QRS width. For LOT-CRT pacing, the nominal PAV (130 ms) was more than 30 ms away from the optimal PAV for 55.9% (19/34) of patients according to LV dP/dt_max_, and more than 30 ms away from the optimal PAV for 40.0% (10/25) of patients according to QRS width.

For BVP, the best-fixed PAV was 163 ms, with an average error of 33.0 ms from each patient's optimal value. The regression-based approach reduced this error to 22.2 ms. For LBBAP, the best-fixed PAV for all patients was 155 ms, with a 34.6 ms average error, which was reduced to 26.6 ms using the regression-based approach. For LOT-CRT, the best fixed PAV was 163 ms, with a 29.2 ms average error, which was reduced to 19.3 ms using the regression-based approach.

### Optimal VV delay

**Figure 6:**
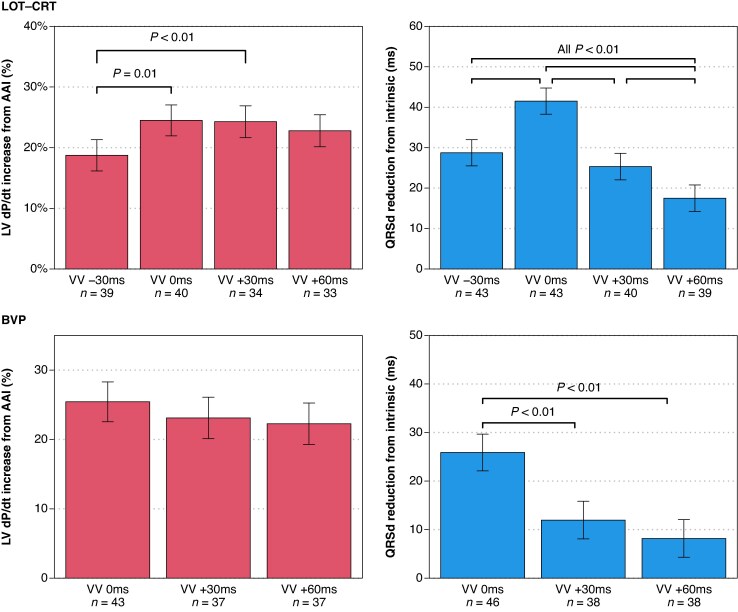
The influence of VV delay when AV is fixed at AV70%. VV delay is listed as the time relative to LVcv stimulation (e.g. ‘VV +30 ms’ indicates LVcv stimulation first and either LBBAP or RV stimulation delayed by 30 ms). Top: During LOT-CRT, LV dP/dt_max_ was significantly decreased when LVcv was paced 30 ms after LBBAP, and the greatest reduction of QRS duration occurred with simultaneous pacing. Bottom: During BVP, no significant differences in LV dP/dt_max_ were observed, but the greatest reduction of QRS duration occurred with simultaneous pacing. Error bars represented the model-based estimate of the standard error of the mean.

*Figure 6* summarizes the average impact of VV delay on LOT-CRT (upper panels) and BVP (lower panels), with AV delay held constant at AV70%. For LOT-CRT, pacing LBBAP first by 30 ms significantly reduced the improvement in dP/dt_max_ compared to simultaneous pacing (*P* = 0.01) and to LVcv-first by 30 ms (*P* < 0.01). QRS duration was minimized with simultaneous pacing, which outperformed all other VV delays. Similar patterns were observed with BVP (lower panels). For QRS duration, simultaneous pacing resulted in a significantly greater reduction than the other tested VV delays.

## Discussion

Our findings suggest that LBBAP, LOT-CRT, and BVP have similar sensitivity to AV delay optimization. A 60 ms deviation from the patient's optimal AV delay reduced the haemodynamic benefit by approximately 30%. While optimal AV delay for BVP and LOT-CRT was similar across haemodynamic and electrical measures, LBBAP preferred a shorter AV delay for haemodynamic benefit than for electrical resynchronization. The correlation between intrinsic AV conduction and optimal AV delay supports a patient-specific approach to AV-timing (setting PAV to approximately 54%–56% of the Ap—LBBs time for LBBAP and LOT-CRT), which outperformed both the nominal and the best-fixed AV delays. Finally, a VV delay of 0 ms was optimal for both LOT-CRT and BVP.

Both haemodynamic and electrical metrics showed that AV delay optimization was similarly important for BVP, LBBAP, and LOT-CRT (*Figures 2* and *3*). In this study, all patients had preserved AV conduction, requiring AV delay optimization to balance both optimal fusion between paced and intrinsic ventricular activation and adequate ventricular filling, which can be compromised by overly short or long AV delays.^1,13^ This balance appears equally important for BVP, LBBAP, and LOT-CRT. The results of this study cannot be extrapolated to patients with AV block.

LBBAP benefited from shorter AV delays than BVP or LOT-CRT, although this was evident for LV dP/dt_max_, not QRS duration (*Figure 4*). In contrast, BVP and LOT-CRT had similar optimal delays by both measures. *Figure 5*, which integrates both metrics, also suggests shorter AV delays are preferable for LBBAP. Liang et al. similarly reported shorter AV delays for LBBAP than BVP, with optimal delays for LV dP/dt_max_ and systolic blood pressure shorter than those for QRS duration.^14^ Theoretically, this is to be expected because successful LBBAP relies on pre-excitation of the conduction system before myocardial activation.

In BVP, the interval between atrial events (paced or sensed) and RV-sensed events has been used to tailor AV delays.^15^  *Figure 5* shows that a similar strategy using an atrial event and LBBAP sensing can personalize AV delays in LBBAP and LOT-CRT. Two independent studies support our findings. Liang et al. studied 21 patients with heart failure and LBBB and found an Ap-QRS interval of 229 ± 44 ms, with an optimal PAV for LBBAP (according to LV dP/dt_max_) of 134 ± 24 ms, yielding a ratio of 58.5%, which closely aligns with our regression slope of 54% (*Figure 5*, middle).^16^ Du et al. studied 21 patients with LBBB and LVEF < 35%, with LBBAP,^17^ reporting an As-RVs interval of 156 ± 33 ms and an optimized SAV (according to a mixture of QRS duration and echocardiographic measurements) of 102 ± 10 ms, a ratio of 65.4%, again near our regression slope. We found that Ap-RVs intervals (279.0 ± 49.2 ms) were nearly identical to the Ap-LBBs intervals (278.4 ± 48.1 ms, *P* = 0.85). This potentially counterintuitive result may reflect the conduction disease in our cohort (i.e. a high proportion of IVCD) or may result from the LBBAP lead position (DSP rather than LBBAP).

One AV optimization strategy is to apply a fixed delay to all patients, represented by horizontal lines in *Figure 5*. In contrast, linear regression models provide individualized delays. Compared to the best fixed delay, patient-specific delays based on the intrinsic AV interval reduced the average error by about 10 ms. For comparison, Whinnett et al. showed that deviations from optimal AV delay reduce systolic pressure, with greater effects at higher heart rates.^18^  *Figure 5* includes patients with long (atrial paced) PR intervals, some exceeding 400 ms. While AV synchrony concerns might suggest limiting maximum PAV, neither LV dP/dt_max_ nor the QRS duration indicated a need to limit the maximum PAV. Other metrics of cardiac function, such as stroke work^19^ or diastolic function,^20^ may better define an upper limit. Excluding patients with long (atrial paced) PR intervals would have resulted in smaller values of the best-fixed PAV, potentially aligning with nominal CRT settings (130 ms).

Our results support a VV delay of 0 ms as optimal for LOT-CRT and BVP (*Figure 6*). For both LOT-CRT and BVP, pacing LVcv first appears to have minimal consequences for LV dP/dt, but QRS duration shows a stronger preference for simultaneous pacing. The results may differ in patients with more marked latency. Using a validated computer simulation of LOT-CRT, Strocchi et al. showed that LBBAP-first was best if the pathology included conduction system delay, and LVcv-first was best if the pathology included intra-myocardial delay.^21^ Our cohort included 40% LBBB and 60% IVCD, which may explain our population's preference for zero VV delay during LOT-CRT. Our preference for zero VV delay during LOT-CRT also suggests that the ‘conduction delay’ following LBBAP (either for propagation from the LV septum or for propagation down a diseased conduction system) likely matches the ‘conduction delay’ in LV free wall activation following epicardial LV pacing, to result in a beneficial fusion. For comparison, Perego et al. and van Gelder et al. found that BVP with LVcv paced 24–25 ms before RV pacing maximized LV dP/dt,^22,23^ whereas our findings (*Figure 6*) show no significant difference between zero VV delay and LVcv paced 30 ms before RV pacing. The differences in findings between our work and that of Van Gelder et al. may result from differences in the haemodynamic acquisition protocol. In the present study, we used a protocol of multiple repeated measurements to minimize the impact of noise. Finally, comparing *Figures 2* and *6* suggests that during LOT-CRT, AV delay appears to have a greater impact on haemodynamics than VV delay, consistent with findings by Whinnett et al. for BVP.^2^

### Limitations

Measurements of QRS width and LV dP/dt_max_ were conducted by a single reviewer (DK) who was not blinded to pacing configuration. The sequence of pacing configurations was not randomized; however, the atrial pacing rate was held constant throughout the procedure, and LV dP/dt_max_ was always measured relative to neighbouring AAI pacing segments, to mitigate any effects of drift. Fitting a parabolic model to a patient's AV sweep and selecting the vertex as the best AV (*Figure 1*) required the exclusion of patients with poorly fitting models or flat responses from *Figures 4 and 5*, which may limit generalizability. We could not identify unique patient characteristics of this excluded patient group. Patients who were relatively insensitive to AV-timing will also be insensitive to any method of AV optimization. All patients had some intrinsic conduction via the AV node in sinus rhythm; therefore, conclusions about AV optimization may not apply to those with complete heart block or AF, where fusion is not possible. Additionally, the cohort was biased toward IVCD over LBBB. Although multivariate analysis found no significant effect of conduction type on optimal AV delay, the study was not adequately powered to draw conclusions about subgroups. Our pacing protocol used overdrive atrial pacing to maintain heart rate during repeated LV dP/dt_max_ measurements, providing data directly applicable to PAV. While the relationship between the best SAV and the atrial sensed to ventricular sensed interval likely parallels the PAV relationship (*Figure 5*), this was not formally validated. Atrial overdrive pacing (∼10 BPM above sinus rate) is known to prolong the AV interval. However, because both the determination of optimal AV delay and the measurement of the Ap-RVs or Ap-LBBs intervals were performed under the same conditions, the influence of overdrive pacing should be minimal. Our relationship between the best PAV and the atrial paced to ventricular sensed interval (*Figure 5*) was based on equal contributions from electrical synchrony (i.e. QRS width) and mechanical synchrony (i.e. LV dP/dt_max_). Although we provided no specific justification for this combination, we found that the use of LV dP/dt_max_ alone produced similar results. For LBBAP-only, the use of only LV dP/dt_max_ provided a linear fit y = 0.51 × + 7.1, whereas the combination of LV dP/dt_max_ and QRS width resulted in y = 0.54 × + 3.8. Finally, the approach to optimal PAV timing was developed for standard atrial appendage pacing. With Bachmann Bundle pacing, a similar approach is anticipated because Ap–LBBs conduction times and optimal PAV intervals are expected to shorten. However, additional study is needed to validate that assumption.

### Conclusion

BVP, LBBAP, and LOT-CRT are similarly sensitive to AV delay optimization, although LBBAP benefits from a shorter optimal AV delay than LOT-CRT or BVP. Patient-specific AV delays, derived from intrinsic AV conduction, outperformed fixed delays in achieving optimal timing. Finally, simultaneous biventricular activation was optimal for both LOT-CRT and BVP.

## Data Availability

The raw data from this study is owned by the funder who wishes to preserve the data for the development of commercial products; however, upon reasonable request, the funder will consider collaborative sharing of data.
